# On the Mechanism of Laboratory Earthquake Nucleation Highlighted by Acoustic Emission

**DOI:** 10.1038/s41598-020-64272-1

**Published:** 2020-04-29

**Authors:** A. A. Ostapchuk, K. G. Morozova

**Affiliations:** 1 Sadovsky Institute for Dynamics of Geospheres of Russian Academy of Sciences, 119334 Moscow, Russia; 2 Moscow Institute of Physics and Technology, 141700, Dolgoprudny, Moscow Region, Russia

**Keywords:** Seismology, Nonlinear phenomena, Characterization and analytical techniques

## Abstract

Dynamics of granular media is the key to understanding behavior of many natural systems. In this work we concentrate on studying regularities of deformation of a gouge-filled fault. Confined granular layer – model fault – subjected to an external stress may display sudden slip owing to rearrangement of the granular layer. In nature fast slip along a fault results in an earthquake. To understand fault behavior better, we have conducted a comprehensive analysis of acoustic emission (AE) data that accompany stick-slip in granular media. Here we reveal and trace the emergence of two populations of AE. The first one is characterized by a waveform with a harsh onset, while the second one exhibits a gradual amplitude rise and a tremor-like waveform. During a regular stick-slip the statistical properties of the first population remains intact. The second one is very sensitive to alterations of stress conditions, and its scaling parameters correlate with the change of mechanical characteristics of the fault. Probably, AE populations were identified corresponding to two gouge-filled fault subsystems – a load-bearing granular network and an ensemble of relatively unloaded grains in the granular layer. The detected regularities point to a compound self-organization processes in fault zones and suggest that the final stage of earthquake preparation can be revealed in analyzing the scaling characteristics of seismic-acoustic data.

## Introduction

Granular media play a key role in a wide range of problems, including self-organized critical behavior, friction in granular flows, geophysical aspects of soil liquefaction, fault dynamics, industry problems of effective treating granular materials, etc.^1–8^. Great attention is paid to factors that determine spatially heterogeneous structure of the granular media, force distribution, jamming transition, etc.^9–16^. Under shear even a slight change of granular packing may lead to a radical alteration of physical properties and mechanical behavior of the granular medium^17–19^.

Investigating the dynamics of granular media helps to deepen our understanding of the process of earthquake nucleation. The concept that frictional instability is the most likely mechanism of shallow earthquakes is now dominating^20,21^. The dynamics of frictional behavior of a block-fault system under normal and shear stresses is investigated in numerical and laboratory experiments to understand the laws of earthquake preparation^16,22–30^. A block-fault system is presented by two solid blocks separated by a layer of granular material^31^. The simplest regime of stick-slip characterized by a slow buildup and a more rapid release of accumulated stress in the system is considered to be an analogue of the seismic cycle^20^. Time variations of mechanical characteristics of a block-fault system were studied in detail. It was shown that as the system approaches slip instability a decrease of fault stiffness is observed^32,33^. The transition to a critical state is accompanied by a number of seismic-acoustic effects^33–36^.

In order to understand, forecast and control the emergence of a dynamic instability on a gouge-filled fault, it is important to detect and trace the physical mechanisms from the lowest scale, the grain size, where instability nucleates, to the scale of the system, where the loss of stability can be noticed. It was shown that zones of stress localization emerge in a laboratory fault under normal and shear loads – force chains, whose ensemble forms the load-bearing framework^16,17,37,38^. The evolution of the framework accompanied by AE governs the emergence of dynamic slip events. AE experiments can reproduce qualitatively the main statistical laws describing seismicity (Gutenberg- Richter power law, Omori law, inverse Omori law)^39–42^. Other qualitative similarities with dynamics of seismicity can also be detected, for example, variation of sliding regime, seismic quiescence before “strong” events, variations of scaling properties of seismicity^34,43–46^. Despite an available groundwork in understanding the dynamics of a block-fault system, interpreting the data of seismic and geoacoustic monitoring is often impeded or impossible at all, and earthquake forecast turns to be ineffective^47,48^.

Here we present laboratory experiments directed towards a comprehensive investigation of AE in the course of shear deformation of a laboratory block-fault system. A thorough consideration of AE waveforms has allowed to detect two populations of acoustic pulses. An analysis of temporal variations of scaling parameters of each population allows to speak about the formation of a “fine structure” of the process of laboratory fault self-organization and demand reconsidering the point of view on acoustic manifestations of the process of preparation of dynamic fault instability.

## Results

**Figure 1:**
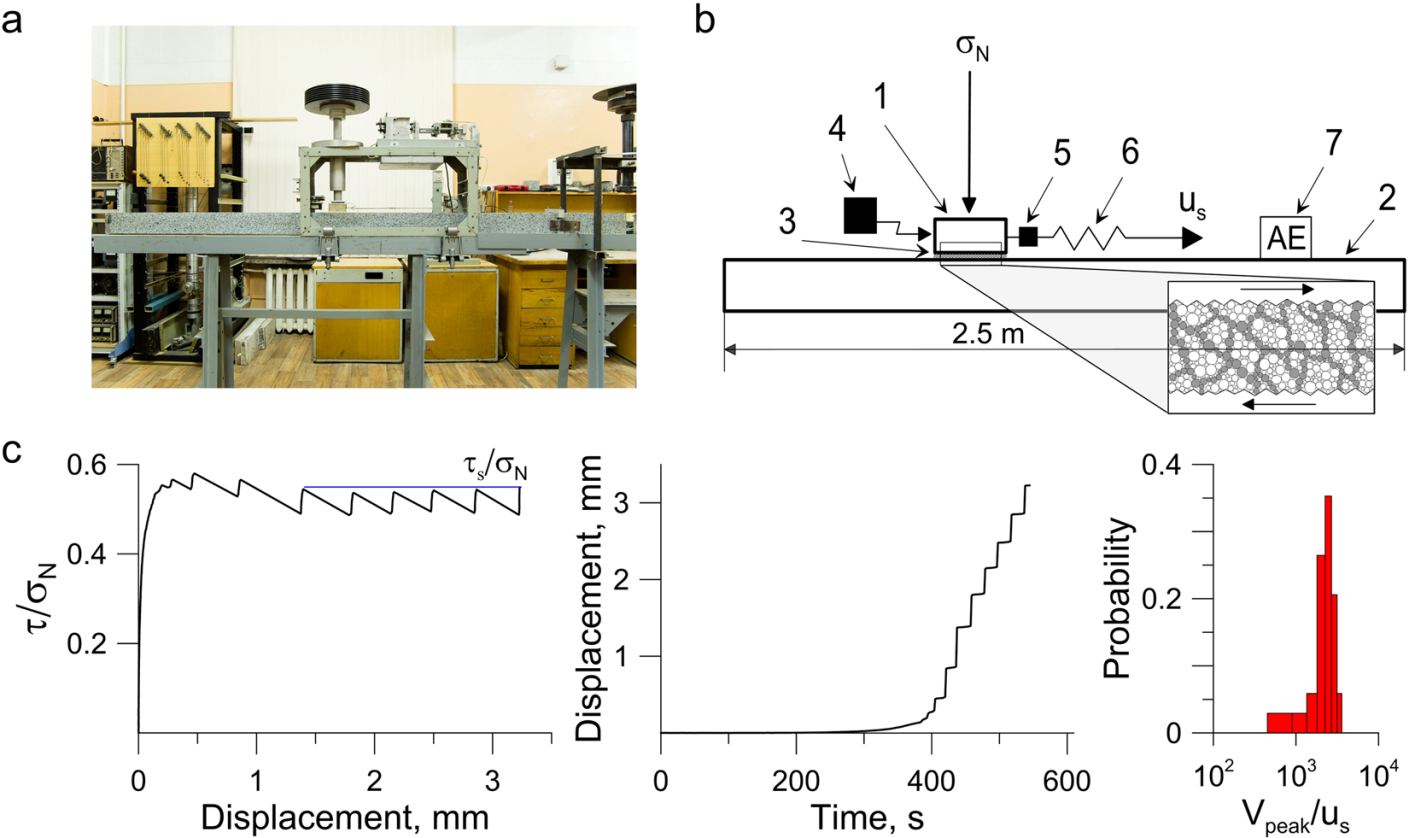
Shear test performance. Experiments were held in the statement of a 1D slider model at the set-up shown in the photo (**а**) and in the scheme (**b**) 1 - moveable block; 2 - granite rod; 3 - gouge layer; 4 - displacement sensor; 5 - force sensor; 6 – spring element; 7 - AE sensor. The inset (figure b) shows schematically the self-organization processes that take place in the thin granular layer^16,17,52^. (**с**) Frictional and kinematic data recorded during the experiment No. 2. The stick-slip regime regularized over a few millimeter before reaching steady state. At the stage of ‘mature’ fault dynamic slip events realize with close parameters.

Experiments on the slider-model are a widely used 1D analogue of a block-fault system^31,49,50^. During the experiment the block under normal and shear loads slides on the base (Fig. 1a,b, Supplementary Figure S1). The contact gap between them is filled with a layer of granular material. In such experiments the elasticity of the loading system controls accumulation of the energy of elastic deformation, similar to the enclosing rock massif in nature. While the dynamics of block behavior is governed by self-organization processes that take place in the interface and by the rheological properties of granular materials^27,30,51^. An important peculiarity of our set-up is the base made of a granite rod 2.5 m long. This allows the AE sensor to be positioned at a distance of 50 cm from the model fault 8 cm long and consider acoustic manifestations of the block-fault system dynamics in the “far-field zone” of the source of vibrations. In previous studies, AE sensors were usually located in the “near-field zones”.

In our experiments we realized the stick-slip behavior at the model fault, which was achieved by using a finely divided granular filler^26,53^. Properties of the model fault alter continuously during stick-slip. A typical view of temporal variations of the mechanical parameters is presented in Fig. 1c. The macroscopic manifestation of frictional instability during stick-slip is the fast slip. A mechanical steady state is observed after the fault residual strength (τ_s_) is reached. This mechanical steady state exhibits relatively uniform intervals between slip events and uniform amplitudes (Fig. 1c). The laboratory set-up and the experimental technique are described in detail in the section “Method”, and the parameters of realized regimes – in the Table S1 of the Supplementary Material.

**Figure 2:**
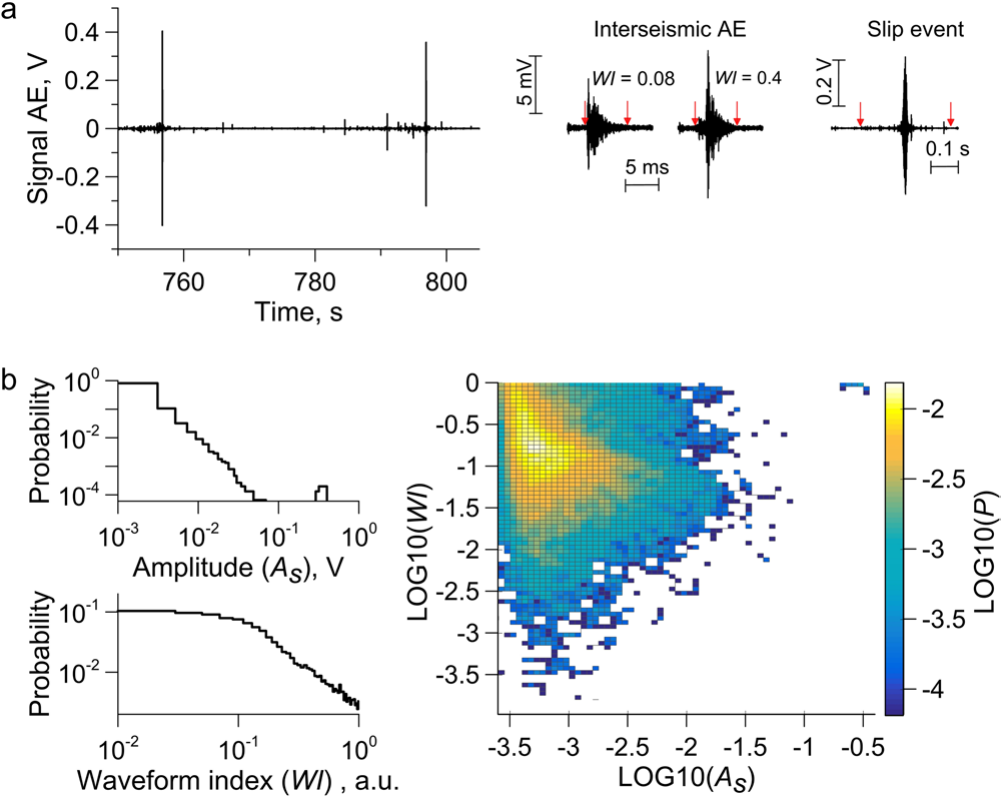
AE data (Exp. No. 5 – Share of glass beads 20%). (**a**) Numerous AE with various waveforms are emitted during a laboratory seismic cycle. AE with maximum amplitude correspond to dynamic slip. The *WI*-value serves as the characteristics of AE waveforms. (**b**) The AE statistics demonstrates an essential difference between distributions over amplitude and over the *WI*-value. Amplitude-frequency distribution is described by a power law of Gutenberg-Richter^39^. In the waveform index plot the characteristic value of *WI* = 0.1 can be detected. In the area *WI* ≤ 0.1 a uniform distribution of AE pulses is observed, while in the area *WI* > 0.1 a power decrease takes place, the slope of the curve being equal to the w-value (relation 5, Method section). Two-dimensional distribution of the AE amplitude and *WI*-value doesn’t expose correlation between AE parameters.

Deformation of the model fault is accompanied by emission of high-frequency acoustic pulses of various amplitudes. We evaluated AE waveforms and detected both pulses with distinct onsets and tremor-like signals at the stage of preparation of dynamic failures (Fig. 2a). We used the waveform index (*WI*-value) as the parameter that characterizes the waveform of the acoustic pulse (see section “Method”). Typically, the *WI*-value ranges from 0 to 1 and indicates the relative location of the peak-to-peak amplitude moment.

Distributions of AE over energy, amplitude, duration and recurrence interval have similar power type, so these parameters can’t be treated as independent ones. The AE statistics over *WI*-value differs essentially from the distributions over amplitude (Fig. 2b). Amplitude-frequency distribution is a superposition of two constituents: a truncated power law distribution (Gutenberg-Richter type) of low amplitudes and a peak-like distribution of high ones. At the same time, the AE distribution over the *WI*-value exhibits two specific areas: *WI* ≤ 0.1 and *WI* > 0.1. In the area of *WI* ≤ 0.1 (harsh onset) a uniform distribution is observed, while in the area of *WI* > 0.1 (gradual amplitude rise) a power distribution manifests. Consideration of the two-dimensional distribution of the AE indicates that there are no correlations between the AE parameters (Fig. 2b). This essential difference points to the thing that the *WI*-value can serve as an independent indicator of the internal processes of fault dynamics. It should be noted that the AE’s waveform together with the rise time/amplitude value point to the fracture mode^54,55^.

**Figure 3:**
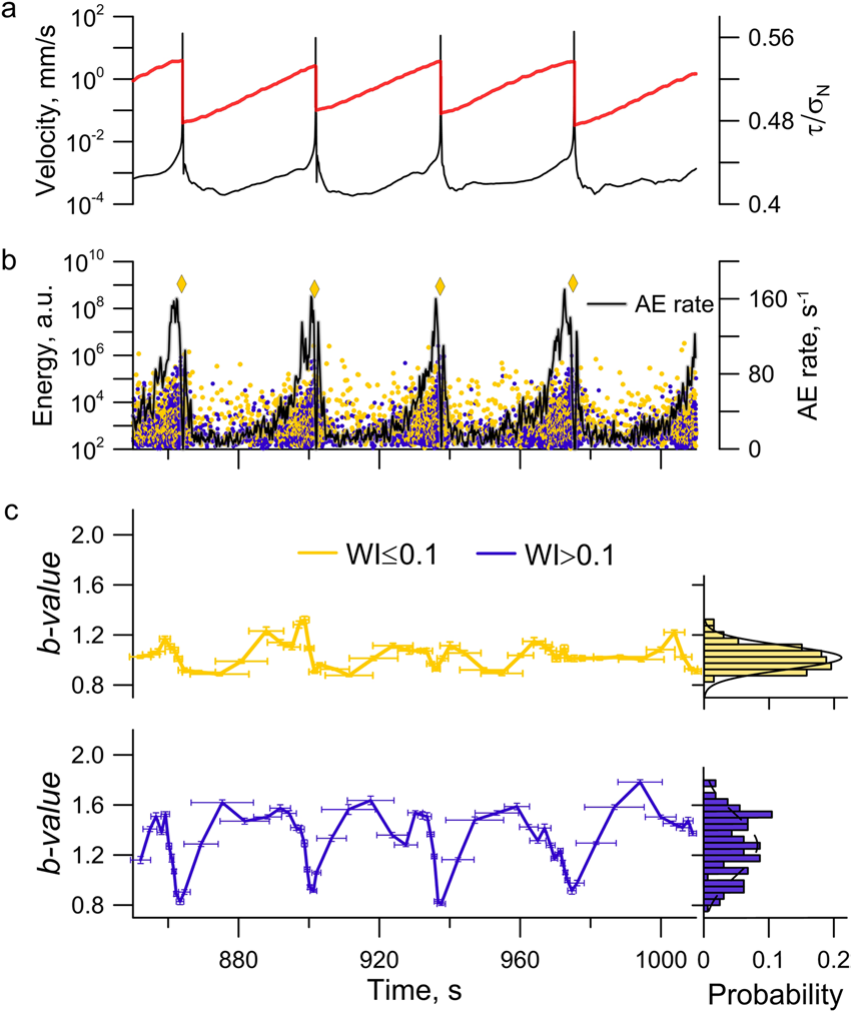
Variations of mechanical and statistical parameters of AE during laboratory seismic cycles (Exp. No.5 – Share of glass beads 20%). (**a**) Time variations of block velocity (black) and friction (red) at the stage of steady state. (**b**) Variations of AE rate are shown with the black line. The AE is combined of two populations: *WI* ≤ 0.1 (yellow) and *WI* > 0.1 (blue). (**c**) The evolution of *b*-value for the two AE populations and the histogram of *b*-value for the experiment on the whole (to the right). For *WI* ≤ 0.1 a slight random variation of *b*-value is observed, which is described by a normal distribution law (black line in the histogram). For *WI* > 0.1 the *b*-value mimics the evolution of mechanical characteristics, and the distribution has a complicated view.

The detected peculiarities of AE distribution over *WI*-value probably testify the formation of two different populations of AE (*WI* ≤ 0.1 and *WI* > 0.1). In order to reveal the “fine structure” of internal self-organization processes we have investigated the evolution of statistical properties of each of the populations (see section “Method”). Considering mechanical behavior of the fault and acoustic data together provides a comprehensive view on the evolution of fault dynamics during a laboratory seismic cycle (Fig. 3).

The analysis of mechanical characteristics clearly testifies the presence of three distinct phases of dynamic slip event preparation, the, so called, post-seismic, inter-seismic and pre-seismic stages of the seismic cycle. At the first stage, just after the dynamic slip, recovery of the fault takes place – deceleration of block sliding is observed, in spite of the fact that the applied shear load grows. Fault recovering is accompanied by a power decrease of AE rate^40,42,46^. Then the interseismic stage starts, which is associated with the elastic load of the fault. The fault is considered to be locked at this stage, the only difference is that the sliding velocity is close to, but not equals zero. As the shear load grows the deformation becomes non-linear, which leads to an acceleration of sliding^56^. The phase of accelerated sliding and power growth of AE rate indicates coming of the third stage, the pre-seismic one^34,46^, which ends with the dynamic slip event, its peak velocity exceeding the velocity of external loading many times (Fig. 1c).

Figure 3c shows regularities of variations of *b*-value of two AE populations. During stick-slip the population of AE with *WI* ≤ 0.1 exhibits approximately constant *b*-value and its variations are random. For the AE with *WI* > 0.1 variations of *b*-value are of a systematically repeated character. Fast growth of *b*-value is observed after the dynamic slip event at the first stage of fault recovering. Then, at the stage of elastic loading, *b*-value remains approximately constant, which manifests in emergence of a peak in the *b*-value histogram in the vicinity of the value of 1.4. At the final pre-seismic stage of the seismic cycle, a monotonic decrease of *b-*value is observed, i.e. the share of high-amplitude AE grows. It should be noted that for the population of AE with *WI* ≤ 0.1 maximum AE amplitude remains approximately the same during the seismic cycle, while for the population with *WI* > 0.1 maximum AE amplitude increases gradually as the system approaches failure (Fig. 3b). The revealed regularities are characteristic for all the performed experiments (see Supplementary Figures S2–S10).

An essential difference of temporal variations of scaling parameters of the two AE populations points to the presence of a “doublet” structures, which develop simultaneously. Preservation of scaling invariance is observed for one of the structures, while for the other one variation of scaling parameters correlates with alteration of fault stress conditions.

## Discussion

Constructing experimental and theoretical links between AE statistics, fault dynamics and internal self organization bases on a large number of various investigations. Formation of a load-bearing granular network and its influence on fault behavior has been observed in many studies^9,16,17,37,38,52,53^. We think that an analogous load-bearing granular network does form in our experiments too (inset in Fig. 1b). So, our experiments probably provide an insight into the structural evolution of a gouge-filled fault and rearrangement of a confined granular layer subjected to shear.

Considering the blocky structure of a natural rock massif many authors note that stress conditions of a rock massif manifest an essential spatial variability. Clustering of abnormally stressed zones is observed, and the distribution of stresses inside separate structural forms can exhibit a partially inhomogeneous character^57,58^. Actually there are loaded consolidated blocks and relatively unloaded free blocks^59^. Reconstruction of stress state before powerful earthquakes has shown that the rupture developed in the area of the maximum stress gradient^60,61^. So, it is possible to find certain similarities in the evolution of both natural and laboratory faults.

In our work we, probably, succeeded for the first time to understand peculiarities of evolution and self-organization of fault subsystems. The available data testify the emergence of 2 subsystems in the fault central zone during shear. Structural properties of these subsystems are to a great extent controlled by the granular composition of the filler. The more uniform the filler is (and the closer the grains to spheres in their shape are), the higher is the structuredness of the emerging load-bearing framework^26,37,53^. It is the formation and failure of load-bearing granular network that govern the emergence of dynamic slip events. Certain structural and strength characteristics are intrinsic for the consolidated zone^51,62^. Rising shear stress causes step-by-step failure of separate load-bearing chains at a more and more higher scale, and total replacement of destroyed chains and formation of a new force network occurs only during the dynamic slip events^16,17,37^. Destruction of loaded force chains, when an abrupt drop of local stresses occur, is accompanied by emission of a time-sharp impulse^16^. As shear load grows destruction of separate force chains leads to a decrease of the force skeleton stiffness. The decrease of the force skeleton stiffness, which is actually the fault stiffness, vividly manifests in alterations of amplitude and spectral characteristics of seismic vibrations^33,35,36^. In spite of destruction of separate force chains the structuredness of the force skeleton as a whole should be preserved. Taking into account regularities of *b*-value evolution (Fig. 3c) we think that the AE population with *WI* ≤ 0.1 describes the dynamics of load-bearing granular network. The performed analysis of *b*-value variations for the AE population with *WI* ≤ 0.1 testifies only a single stationary state of the corresponding population (Fig. 3c). It means that the structuredness of the load-bearing granular network does preserve. The higher the share of glass beads is (regular spherical shape), the higher is the structuredness of the force skeleton, and, consequently, the higher are the scaling characteristics of corresponding AE population. Increasing the shear of glass beads in the presented experiments is accompanied by an increase of the mean *b*-value for AE population with *WI* ≤ 0.1 (Supplementary Figures S2-S9), which also points to correspondence between the dynamics of load-bearing granular network and the AE population with *WI* ≤ 0.1.

In the unconsolidated areas the action of shear stresses leads to inter-grain slippages and local reconstructions of grain ensembles^62,63^. Obviously, self-organization processes that take place in unconsolidated areas are more gradual, and pulses emitted have higher *WI*-values. As shear stress grows the destruction of separate force chains of the load-bearing granular network leads to an expansion of the sizes of unconsolidated areas, and, consequently, to an increase of the probability of emergence of high-amplitude gradual events (AE with high *WI*-value). In its turn, the increase of probability should be accompanied by a decrease of *b*-value of the corresponding AE population. Taking into account the regularities of *b*-value evolution for the two AE populations (Fig. 3c), we think that the second subsystem of unconsolidated areas corresponds to AE with *WI* > 0.1, and its evolution conforms with the stages of seismic cycle. The subsystem of relatively unloaded unconsolidated grain ensembles has two most probable states (Fig. 3c). The first state corresponds to the stage of elastic / quasi-elastic fault load, when the most probable *b*-value is 1.4, the second state is the final stage of dynamic failure preparation, when *b*-value lowers to 0.8-0.9. Lowering of *b*-value to these characteristic level testifies that the fault has transited to the critical state.

To address the question about how our results relate to fault dynamics in nature, we should note that mechanical behaviors of natural and model faults are qualitatively similar^50^. The main feature of experiments in the slider-model statement is that the energy of elastic deformation cumulates in the elastic unit of loading system, while in nature the energy cumulates in the block adjacent to the fault. At the same time, both in laboratory and in nature the dynamics of energy release is determined by the fault self-organization and frictional properties of fault’s principal slip zone. The main mechanical parameter that governs the sliding regime is the ratio of specific shear stiffness of the fault to shear stiffness of the enclosing rock massif (or stiffness of the loading machine)^27,64^. We admit that the similarity criteria are not satisfied in our investigation, but we believe there is a qualitative correspondence between the processes that take place in natural and laboratory faults. The performed experiments demonstrate that a complex analysis of seismicity and microseismicity could give important information about the fine spatial structure of the fault – formation of the zones, where stresses localize, and relatively unloaded areas. Moreover, findings introduce a perspective direction in studying seismic-acoustic manifestations of the dynamics of fault self-organization – detecting the areas of earthquake nucleation and their transition to a critical state.

## Method

### Slider-model setup

All the experiments were held at the geomechanical bench of IDG RAS in the slider-model statement. A granite or marble block (1) slides along the base (2) under normal and shear loads (Fig. 1b, Supplementary Figure S1). The block size was 8 × 8 × 3.2 cm and the mass − 550 g. The base was a granite rod 2.5 m long and 10 × 10 cm in cross-section. The moveable block was located in the middle of the base. The contact gap between rough surfaces of the base and the block was filled with a layer of granular material 3 mm thick (3). The gouge layer was prepared using the leveling frame, so that the initial thickness of the layer was the same in all the experiments. The maximal change of layer thickness to the end of an experiment did not exceeded 1 mm. The filler was composed of the mixture of quartz sand (grain size 200–315 µm) and glass beads (grain size 100–315 µm). The share of quartz sand was the mass of quartz sand fraction in the filler. All the experiments were performed at room temperature and humidity.

During the experiments the normal stress of σ_N_ = 48 kPa was applied to the upper surface of moveable block through a thrust bearing, which excluded emergence of additional shear stresses at the contact between block and base (Supplementary Figures S1). The normal load was gathered of a number of weights. The shear load was applied to the block through a spring (6) with the stiffness of 55 kN/m. The edge of the spring was pulled at a constant velocity of u_s_ = 8 µm/s. The shear load was monitored with a force sensor (5) CFT/5kN (HBM) with the accuracy of 1 N. Relative block-rod displacements were controlled with a laser sensor (4) ILD2220-10 (Micro-Epsilon) in the frequency range of 0–5 kHz with the accuracy of 0.1 μm.

The stick-slip regime was realized during experiments. A typical view of the loading curve is presented in Fig. 1c. For a detailed analysis we chose the section where the strength of the model fault has reached the residual value of τ_s_ and the dynamic slip repeats quasi-periodically with the same amplitude. At this stage, the model fault is considered to be mature^65,66^.

### AE processing

To monitor the acoustic emission we used the sensor VS30-V (Vallen System) (7), which was mounted on the granite rod at the distance of 0.5 m from the moveable block (Supplementary Figure S1). The sample rate *f*_s_ was 2 MHz. In the course of experiments the operation of loading device and other external acoustic disturbances had a negligible effect on the parameters of the AE signal. The background noise level of AE signals was 50 dB.

The energetic criterion was used to detect AE in the recorded signal - the flow of AE energy should exceed a certain threshold, as follows:

1 $$\varPi (t)=\frac{1}{\Delta t}\mathop{\sum }\limits_{t}^{t+\Delta t}\frac{A{({t}_{i})}^{2}}{{f}_{s}}\ge 1.5{A}_{\min }^{2}$$

where *A*(*t*) is the recorded signal filtered in the frequency band of 20 to 80 kHz, ${A}_{\min }^{2}$ is the variance of the AE signal. The energy flow was determined in a running window with the duration of Δ*t* = 0.5 ms, the running step being Δ*t*/2. ${A}_{\min }^{2}$ was determined in 1-second interval before the loading started according to the variance relation:

2 $${A}_{\min }^{2}=\frac{1}{{f}_{s}-1}\mathop{\sum }\limits_{{t}_{i} > 0}^{{t}_{i}\le 1}{|A({t}_{i})-\frac{1}{{f}_{s}}\mathop{\sum }\limits_{{t}_{i} > 0}^{{t}_{i}\le 1}A({t}_{i})|}^{2}$$

After a preliminary detailed analysis of a number of records and consideration of AE statistics we came to the conclusion that duration of a separate AE pulse should exceed 1 ms. For all the detected pulses the following parameters were determined: onset (*t*_s_), termination (*t*_e_), amplitude (*A*_s_), peak-to-peak amplitude (Δ*A*), energy (*E*), spectral centroid (*f*_c_), and waveform index (*WI*). The *WI*-value was estimated according to the relation:

3 $$WI=({t}_{\max }-{t}_{{\rm{s}}})/({t}_{{\rm{e}}}-{t}_{\max })$$

where *t*_max_ is the moment corresponding to maximum peak-to-peak amplitude. The *WI*-value is similar to the *RA*-value, which determines crack genesis in concrete materials^54,55^. Note that there is no grain cracking in our experiments, AE are generated only in frictional slips of grains.

### AE statistics

Seismic and acoustic activity is often monitored using the well-known frequency-magnitude relation of Gutenberg-Richter^39^:

4 $${\log }_{10}(N)=a-bM$$

where *N* is the number of events with the magnitude not less than *M*, *a* and *b* are two positive constants. The *a*-value is a measure of the seismic activity which depends on the space-time window of observation. The slope *b* (*b*-value) is a scaling parameter which is of critical importance in dynamics of self-organization^1,67^. A relative laboratory magnitude of AE was calculated according to the relation^68^:

5 $$M=\,\log ({A}_{s}/{A}_{0})$$

where *A*_0_ is the threshold for detection.

An essentially different AE distribution has been obtained over the waveform index *WI*-value (Fig. 2b). The change of the share of events with the growth of *WI*-value can be represented as follows:

6 $$N=\{\begin{array}{c}c,WI\le 0.1\\ d\cdot W{I}^{-w},WI > 0.1\end{array}$$

where *N* is the number of AE pulses with the waveform index not less than *WI*, *c* and *d* are positive constants determined by the intensity of AE. The index *w*-value characterizes the non-uniformity of AE ensemble over the *WI*-value, and its alteration points to a change of prevailing mechanism of AE generation.

In order to investigate the temporal evolution of *b*-value, we calculated *b*-values using the method of least squares for a running window of an equal number of events (*nn* = 100) with a running step of *nn*/2 events (50% overlap).

## Supplementary information

Supplementary Information.

## Data Availability

All the data that support findings of this work were collected on geomechanical test bench of the Sadovsky Institute for Dynamics of Geospheres of Russian Academy of Sciences. All the data presented in this study are available from corresponding author on request.
