# Supplementary material for: On the Mechanism of Laboratory Earthquake Nucleation Highlighted by Acoustic Emission
Source: Sci Rep. 2020 Apr 29;10:7245. doi: 10.1038/s41598-020-64272-1 (PMC7190713; doi:10.1038/s41598-020-64272-1)
Supplement: Supplementary file 1 — Supplementary Information. [file 41598_2020_64272_MOESM1_ESM.pdf]

On the Mechanism of Laboratory Earthquake Nucleation Highlighted by Acoustic Emission

[1,2]*Alexey A. Ostapchuk & [1]Kseniya G. Morozova

[1]Sadovsky Institute for Dynamics of Geospheres of Russian Academy of Sciences, 119334, Moscow, Russia

[2]Moscow Institute of Physics and Technology, 141700, Dolgoprudny, Moscow Region, Russia

*Corresponding author: ostapchuk.aa@phystech.edu, ostapchuk@idg.chph.ras.ru

Supplementary Materials

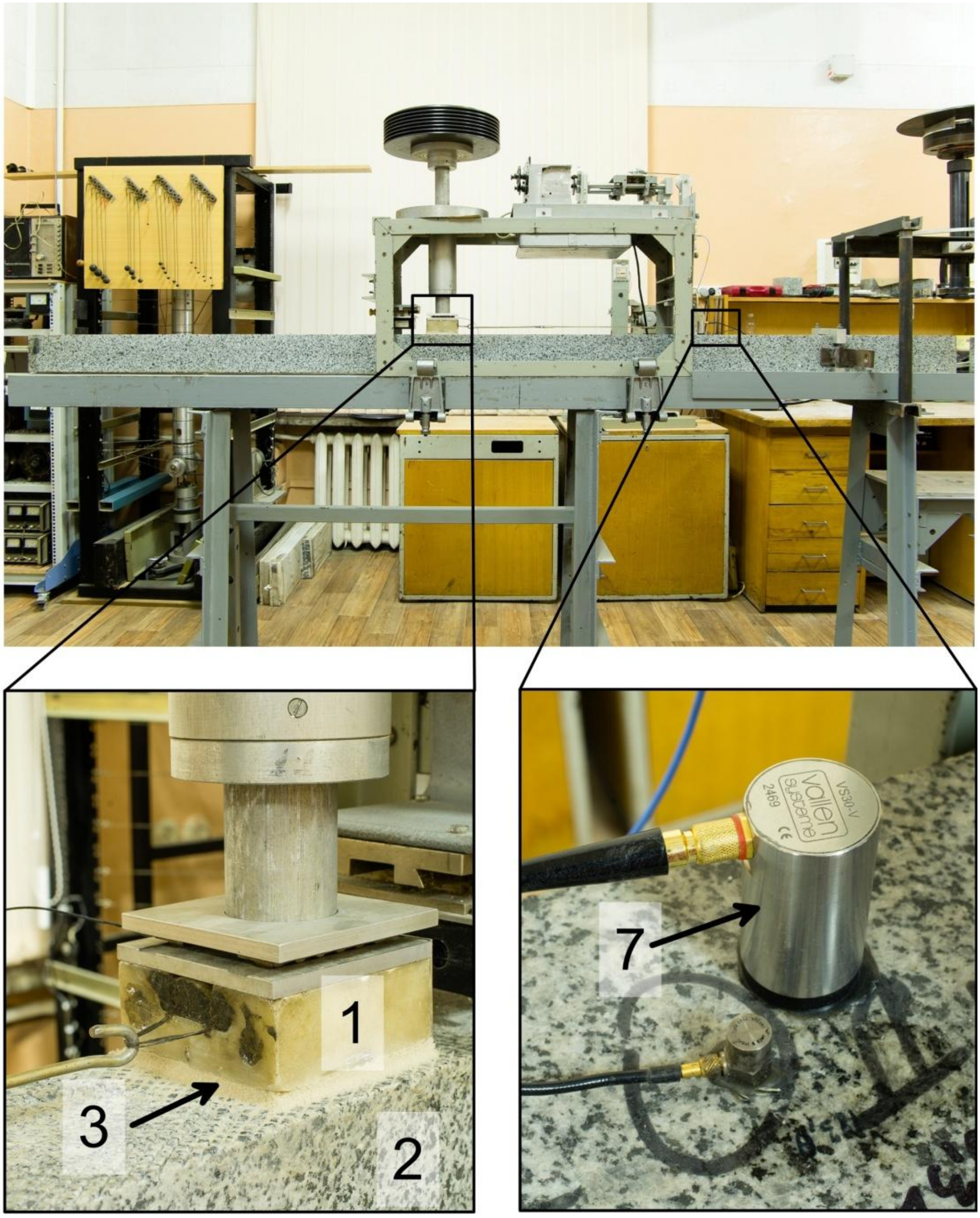

Supplementary Figures S1. The general view of experimental set-up.

(1) moveable block, (2) granite rod; (3) gouge layer; (7) acoustic emission sensor

Exp. No.	Filler, share of glass beads, %	Mechanical parameters of sliding regime			Acoustic emission parameters			
		u_s, μm/s	V_{peak}, mm/s	F_S, N	A_s, dB	min (AE rate), s^{-1}	*b*-value	*w*-value
1	0	8	6.6±1.9	195	102.4±2.6	36±14	1.21±0.04	1.52±0.03
2		8	5.8±1.8	192	99.3±2.7	28±11	1.37±0.06	1.68±0.03
3	10	8	18.1±3.3	167	107.4±1.9	17±8	1.42±0.04	1.55±0.03
4		8	12.9±1.8	179	106.1±1.4	18±8	1.45±0.04	1.60±0.03
5	20	8	26.2±3.8	164	108.7±1.2	13±7	1.39±0.04	1.50±0.03
6		8	20.4±2.8	174	107.3±1.2	14±7	1.49±0.05	1.59±0.03
7	35	8	21.3±3.3	156	104.1±1.4	14±7	1.51±0.04	1.46±0.03
8		8	25.7±3.2	159	105.6±1.6	11±6	1.59±0.04	1.46±0.03
9	50	8	44.0±2.5	144	106.8±1.1	10±7	1.55±0.04	1.48±0.04
10		8	33.0±3.4	141	104.2±1.5	8±5	1.72±0.03	1.37±0.03
11	60	8	21.9±2.0	146	100.8±1.5	6±4	1.79±0.05	1.37±0.03
12		8	24.5±2.7	144	101.6±1.1	5±3	1.67±0.04	1.31±0.04
13	80	8	15.2 ±3.2	141	95.2±1.7	4±2	1.81±0.05	1.30±0.04
14		8	16.2±2.012	141	95.6±1.6	5±3	1.89±0.05	1.28±0.03
15	100	8	15.6±3.5	136	92.4±2.0	4±3	2.27±0.06	1.35±0.03
16		8	14.1±1.3	138	90.7±1.3	5±3	2.23±0.10	1.20±0.04

Supplementary Table S1. Summary of main series' experiments. All test were conducted under the same environmental conditions. u_s – rate of the spring pulling, V_{peak} – peak slip velocity, F_S – fault strength at the stage of ‘mature’ fault, A_s – amplitude of AE, min (AE rate) – minimal value of AE rate at interseismic stage of seismic cycle, *b*-value – parameter in the relation (4), *w*-value – parameter in the relation (6).

Supplementary Figures S2-S9 show the time variations of block velocity and acoustic data for experiments with the different filler. **(a)** block velocity, **(b)** AE rate, **(c)** AE energy, **(d)** *b*-value for AE population *WI*≤0.1 (yellow) and *WI*>0.1(blue).

Below are presented examples of different experiments.

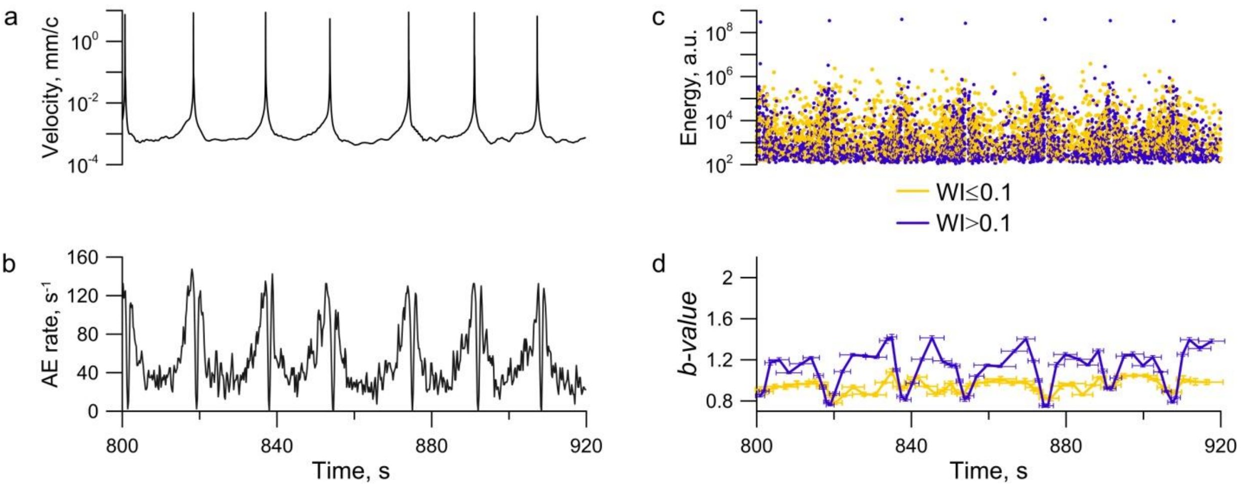

Supplementary Figure S2.Share of glass beads 0 %.

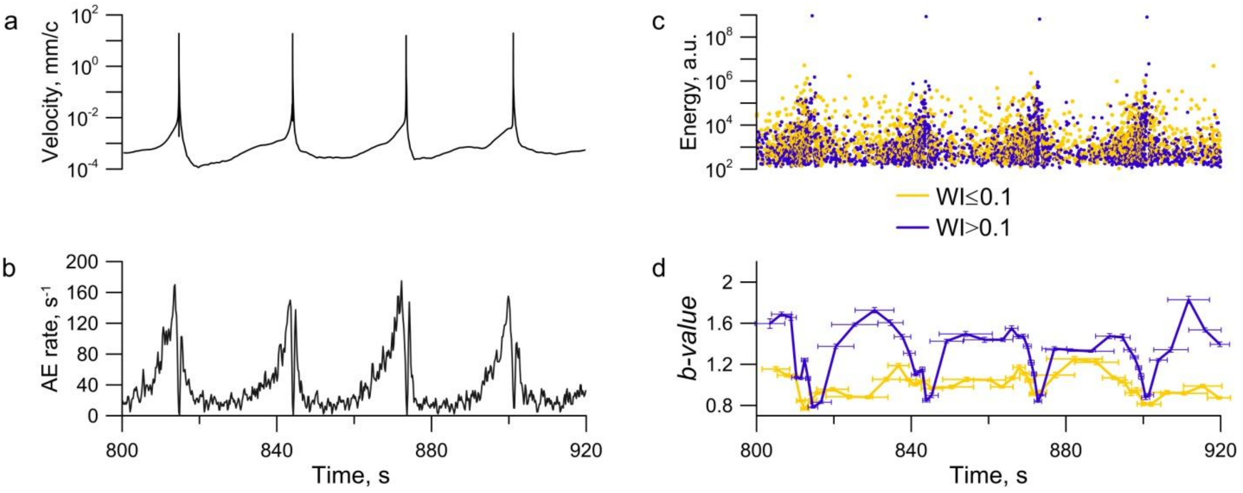

Supplementary Figure S3. Share of glass beads 10 %.

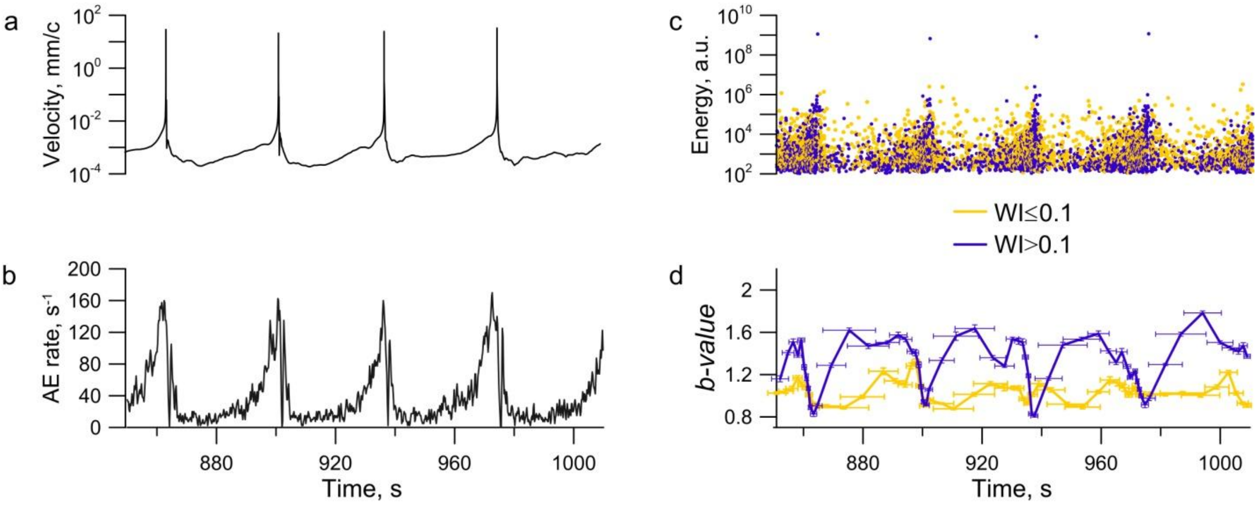

Supplementary Figure S4. Share of glass beads 20%.

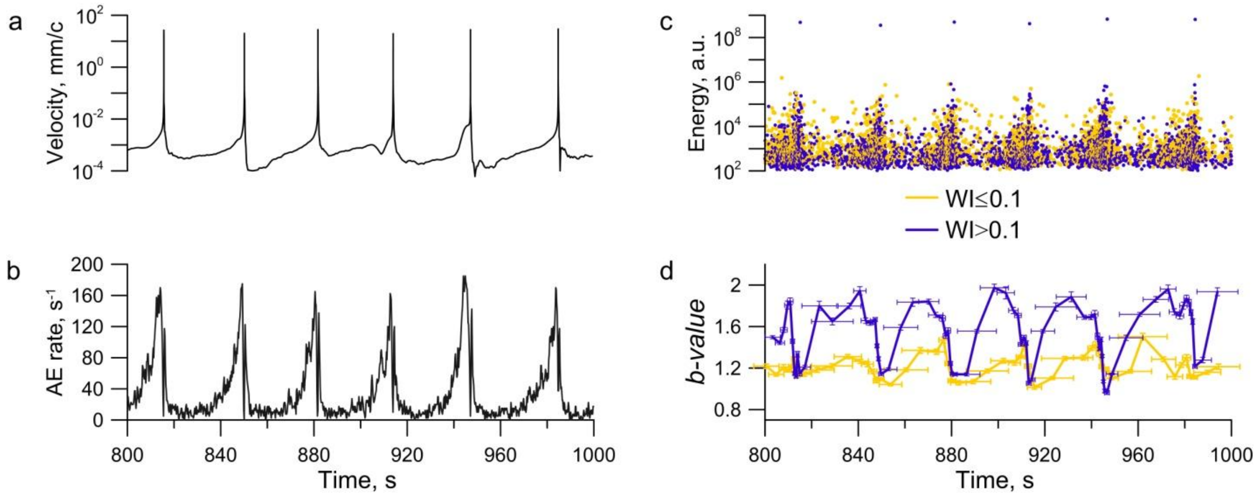

Supplementary Figure S5. Share of glass beads 35 %.

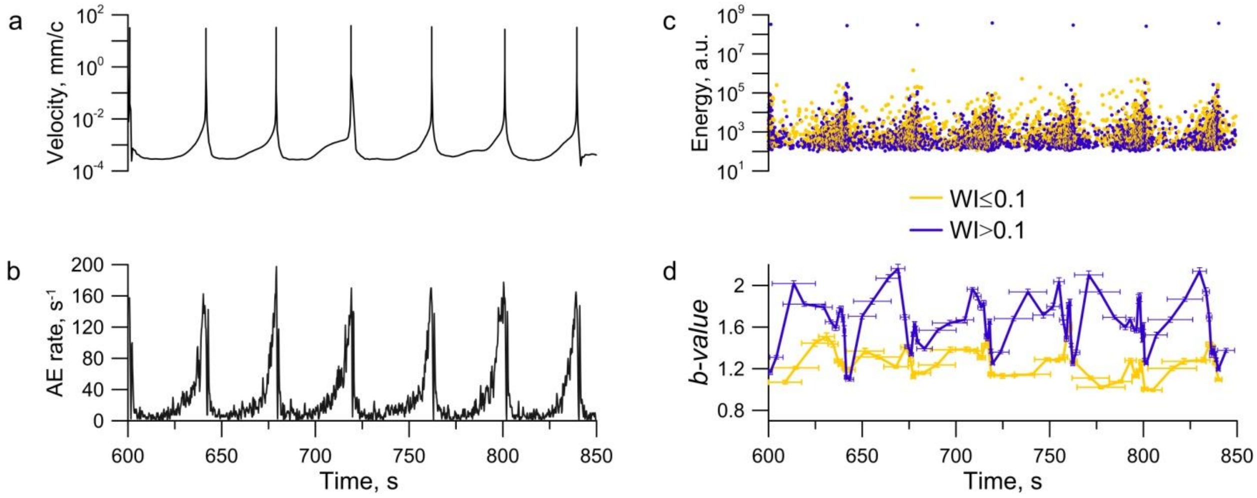

Supplementary Figure S6. Share of glass beads 50 %.

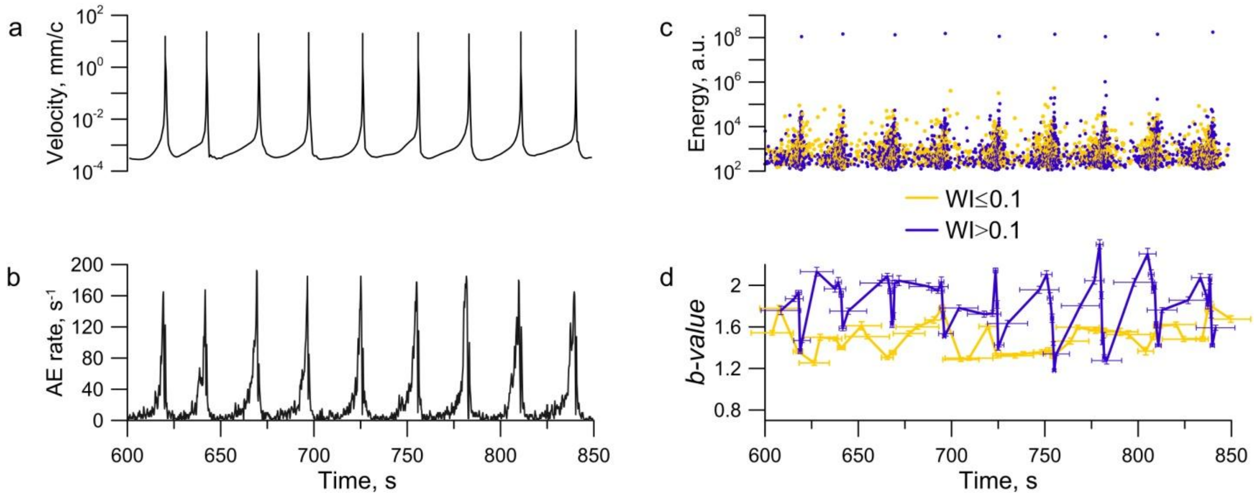

Supplementary Figure S7. Share of glass beads 60 %.

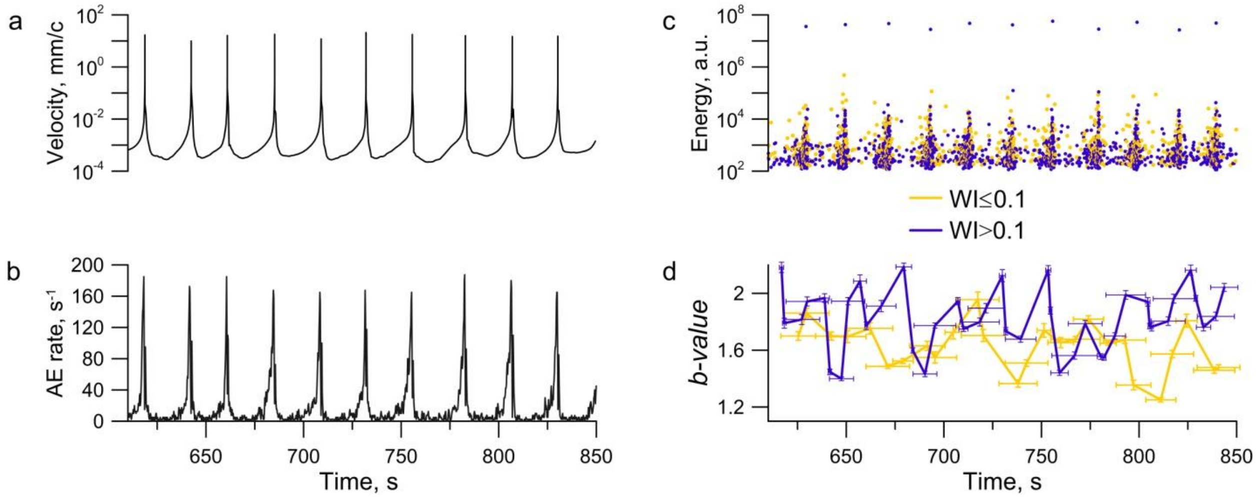

Supplementary Figure S8. Share of glass beads 80 %.

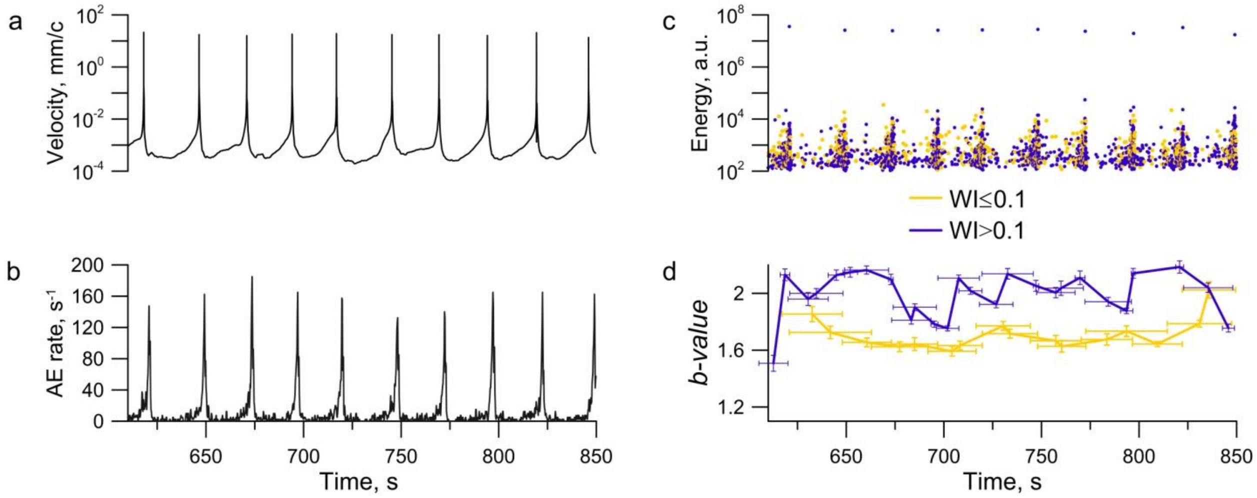

Supplementary Figure S9. Share of glass beads 100 %.

Supplementary Figures S10-S11 show the time variations of block velocity and acoustic data for the extra experiments. **(a)** block velocity, **(b)** AE rate, **(c)** AE energy, **(d)** *b*-value for AE population *WI*≤0.1 (yellow) and *WI*>0.1(blue).

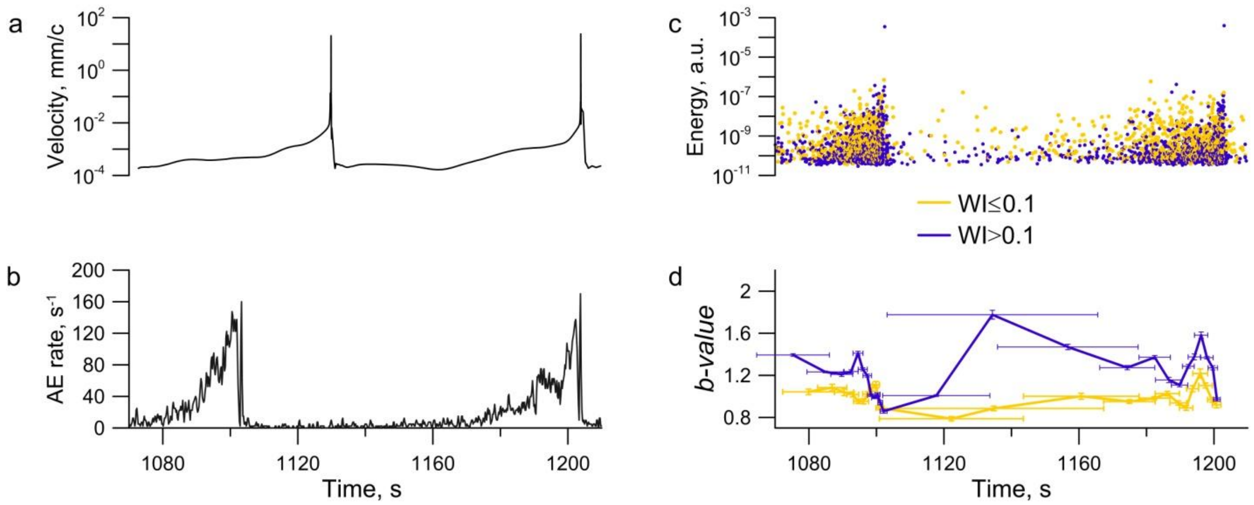

Supplementary Figure S10. Example of high-amplitude stick-slip regime. Filler consists of moistened by water quartz sand

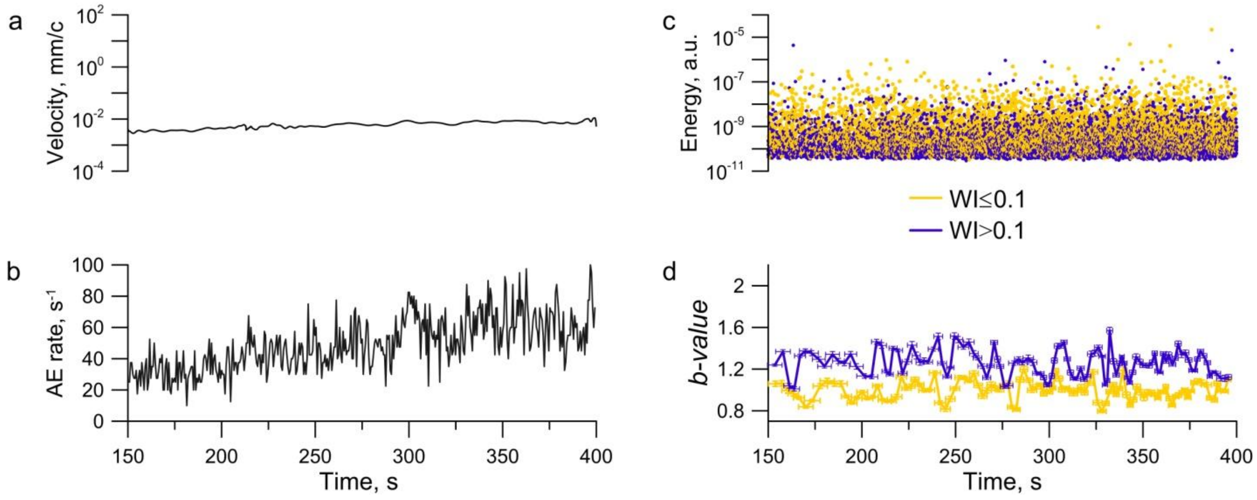

Supplementary Figure S11.Example of stable sliding regime. Filler consists of mixture of quartz sand and kaolin clay share of 30 %.
